# Knapp on the “Cause, Nature, Cure, and Prevention of Epidemic Cholera”

**Published:** 1855-04

**Authors:** 


Prof. M. L. Knapp, late of Iowa University, has an article, in the New York Journal of Medicine, on the "Cause, Nature, Cure, and Prevention of Epidemic Cholera."

Our readers must have had a surfeit of cholera literature. We do not propose to meddle with what Prof. Knapp says of the nature and cure of this disease, but we wish to call attention to his ideas of cause.

We have our own hobby in this matter, but he draws an omnibus of such liberal dimensions as to admit most other theorists to a ride, *gratis*.

After describing the high price of vegetables at Pittsburgh, Pa., at the outbreak of cholera there, he says:

"I visited the markets on the morning of the 26th, and passed through two large and commodious houses, well supplied with choice meats, but not a potato, turnip, tomato, or other succulent vegetable, apple, peach, or other fruit of any description whatever, on sale or to be seen at either market! This is a more forcible commentary on the power and influence of medical opinion for evil than could have been anticipated. It shows very clearly that the prohibition of vegetables and fruits has not been over-estimated by the writer as an unfortunate blunder of the profession, protracting and aggravating every epidemic visitation of the disease.

"Case I. Monday, September 25, 9 o'clock, A. M., through the courtesy of the Board of Health, visited, with Dr. ——, a middle-aged German woman in the fifth ward, in the collapsed stage of cholera, the patient speechless, pulseless, senseless, and cold as in death. The case had had no treatment. On examining the mouth, the objective signs of scorbutus were found most unequivocally manifest in a puffy and livid condition of the gums, pale flabby tongue, especially toward the edges, which bore the impressions of the teeth. This was considered a hopeless case, but the punch, with quinine and morphine added, was prescribed, as the patient was not past swallowing. The case terminated fatally in a few hours, and was only instructive as affording testimony of the presence of the scorbutic diathesis in the patient.

"On examination of the other members of this family, six in number, all showed the objective signs of scurvy in different degrees, by the crimson line along the dental margin of their gums, tongues furred centrally, and pale and smooth laterally, paleness of countenance, inertia of feelings, despondency and dejection of spirits. One suckling woman, in particular, exhibited the signs of the scorbutic diathesis most prominently of all. They were enjoined to drink punch daily, and to make a free use of vegetables and fruits in their dietary."

Following this case he gives minute details of a number of cases. We give an extract from his general remarks:

"The analysis of twenty cases of cholera, then, discloses the fact, that every case was a case of scurvy, not a solitary exception, in, or out of the hospital, comprising *all* seen. This is a remarkable announcement; nevertheless, remaikable as it may seem, every word of it is truth. Had not the physical evidences of scurvy been present in *every* case, I should have marveled. The appearances which I have described will be found in all cases of true cholera, and will henceforth be noticed by all practitioners in all parts of the world. Why they have been so long overlooked, why they should have escaped my observation heretofore, *generally*, and, where noticed in 1849 and '50, a complication of diseases only should have been inferred, is a matter of as much astonishment to me as it can possibly be to others: but such are the facts. Why I have now been made the humble instrument of explaining the matter, is doubtless due to circumstances rather than to extraordinary penetration, or superior professional attainments. It has been a hard and difficult task to divest my mind of the false notion of some specific, poisonous influence, *overlaying* scurvy, even since I have been fully aware of the scorbutic diathesis *underlaying* cholera. It may be difficult for others, even yet, to see clearly: but if, as appears by our analysis, every case of cholera occurs in a scorbutic subject, or in other words, that cholera is a messenger of death riding *always* on the time-honored steed scorbutus, it matters but little what be the theory as to the office or entity of the messenger —if we destroy the steed, the rider will get on but poorly. This we know how to do. But I can see no occasion now to search for further cause of cholera than the causes producing scurvy, no phenomena in cholera other than what harmonize with the known laws of scurvy, and nothing at the bed-side after the hæmorrhagic action is arrested but the physical evidences of scurvy, neither do the books describe any anatomical lesions contradictory to this view."

Now we have no objection to admitting that scorbutus may have a large share in the cause of cholera in some localities. We fully believe that the abstinence from all succulent fruits, so common during cholera years, must be provocative of disease. But if scurvy is the only cause of cholera, why did it never produce it in this country before A.D. 1832? And why did it cause cholera then, rather than in 1833, when vegetables were looked upon as murderous? H.

